# A novel Z-shaped anti-rotation rod for atlantoaxial dislocation reduction: finite element analysis

**DOI:** 10.1186/s13018-025-05723-1

**Published:** 2025-04-29

**Authors:** Mandi Cai, Rencai Ma, Junlin Chen, Xinzhao Huang, Yixing Zhang, Zhuohang Xie, Xiaobao Zou, Xiangyang Ma

**Affiliations:** 1 The First School of Clinical Medicine, Southern Medical University, No.1838 North of Guangzhou Road, Guangzhou, 510515 People’s Republic of China; 2 Department of Orthopedics, General Hospital of Southern Theatre Command of PLA, 111 Liuhua Road, Guangzhou, 510010 People’s Republic of China

**Keywords:** Finite element analysis, Z-shaped rod, Anti-rotation, Atlantoaxial dislocation, Internal fixators, Biomechanics

## Abstract

**Background:**

C1-C2 pedicle screw-rod fixation (PSR) is widely used for atlantoaxial dislocations. However, its limited reduction capacity in refractory cases necessitates additional release surgeries, increasing operative risks including prolonged surgical time and expanded tissue damage. We developed a novel Z-shaped anti-rotation rod to improve reduction capability, but its biomechanical performance requires evaluation.

**Methods:**

A nonlinear atlantoaxial instability three-dimensional (3D) C0-C3 finite element model was constructed using computed tomography images from a 25-year-old healthy male without a history of cervical spine diseases. Based on this model, two C1-C2 fixation configurations were simulated: conventional pedicle screw-rod (PS-CR) and pedicle screw-Z-shaped rod (PS-ZR). Reduction forces were measured and compared. Range of motion (ROM), stress distribution and peak stress values of the implants were recorded and compared under six loading conditions including flexion, extension, lateral bending, and axial rotation.

**Results:**

Both configurations achieved a greater than 98% reduction in the C1-C2 segmental ROM, with similar compensatory motions in adjacent segments. The reduction force of PS-ZR showed significant advantages (2–8 mm range), achieving a maximum reduction force of 88.544 N, which is 1.67 to 3.68 times that of PS-CR. The PS-ZR system experiences greater stress compared to the PS-CR system, escalating with Z-rod height. Regarding stress distribution and peak values of rods, the maximum stress on the PS-CR system was mainly concentrated at the connection between the rod and the screw nut while the maximum stress on the PS-ZR system was concentrated at the transition part of the “Z” shape.

**Conclusions:**

Both PS-CR and PS-ZR configurations provide reliable and comparable stability. Compared to the PS-CR configuration, the PS-ZR configuration provides superior reduction force and stability, potentially reducing the need for additional release surgery and surgical time. This novel design has significant clinical implications for improving fixation techniques.

## Introduction

Atlantoaxial dislocation is a common disorder of the upper cervical spine [1]. Due to the loss of normal anatomical relationships in the atlantoaxial joint, the spinal cord is often compressed. When neurological symptoms such as dizziness, limb numbness and weakness, and even urination and defecation disturbance occur, surgical intervention is usually required to alleviate symptoms and prevent progression [2]. The atlantoaxial joint has a large range of motion, which requires the internal fixation system to achieve adequate reduction, spinal decompression, and stability reconstruction. The posterior atlantoaxial PSR system can provide a rigid fixation without sacrificing the motion of occipitocervical junction and has become the most prevalent fixation technique [3–5].

**Fig. 1:**
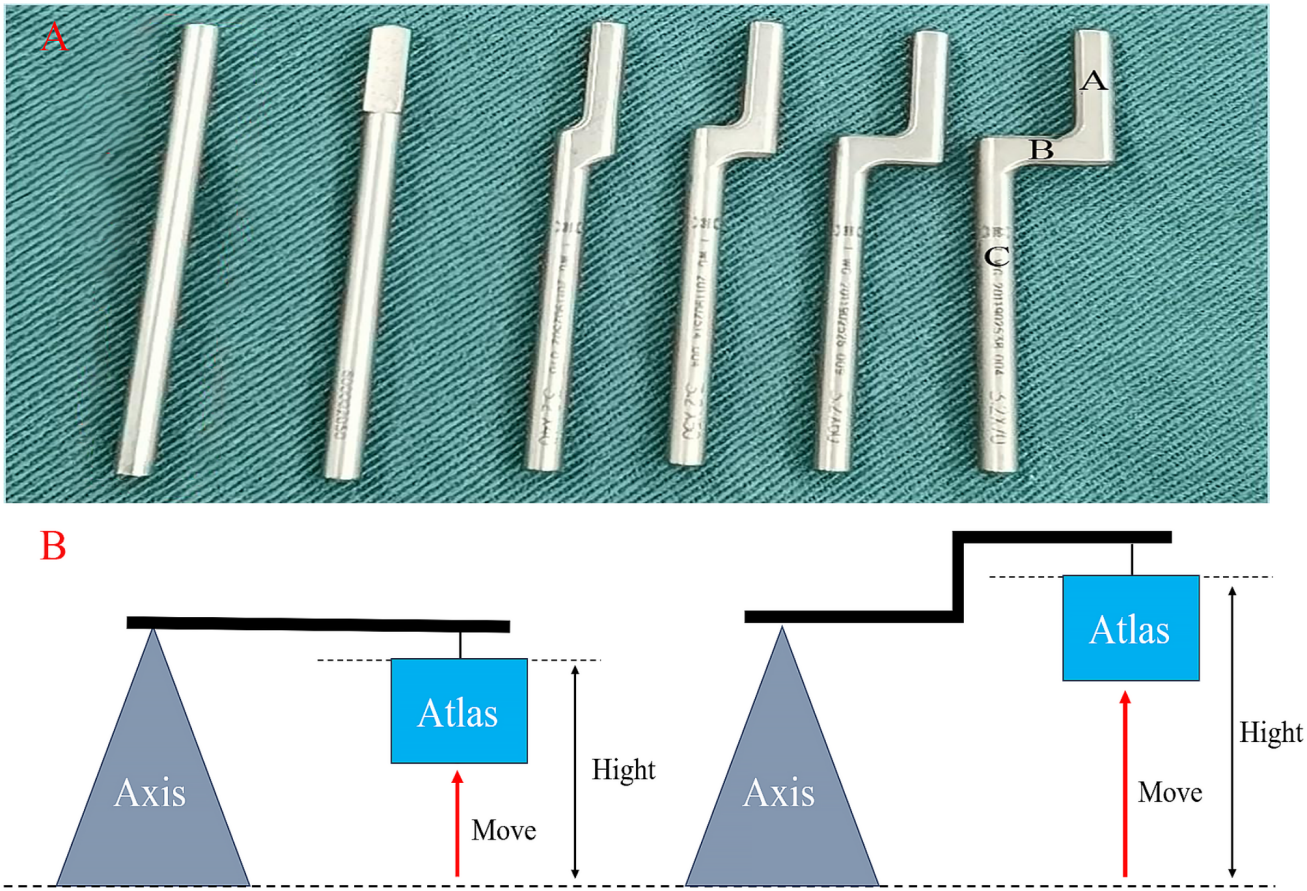
Appearance of novel Z-shaped rod and schematic of lever reduction principle. **1 A**. Comparison of common rod and new Z-shaped anti-rotation rods of different specifications with height intervals of 2 mm from 0 to 8 mm in sequence (black **A** represents the end with a “D”-shaped cross-section, black C represents the end with an “O”-shaped cross-section, and black **B** represents the connecting part). **1B**. The Z-shaped rod increases the height difference between the atlas and axis, leveraging the lever principle to lift the atlas and achieve reduction

The prevalent screw-rod reduction and fixation system mainly relies on the pulling force generated by pre-bent rods. However, their reduction capability is limited, especially for patients with refractory or irreducible dislocations, as it is challenging to achieve reduction solely by relying on bent rods. Meanwhile, excessively bent rods require additional tools for adjustment when placed in the screw slots, increasing surgical time and the risk of dural injury. Based on these issues, we designed a novel type of Z-shaped rods with multi-stage reduction capability and an automatic anti-rotation feature (Fig. 1).

This study aimed to evaluate the advantages of the Z-shaped rod over the common rod in terms of reduction force, ROM and stress distribution of the two internal fixation systems through finite element analysis, providing a theoretical foundation for further clinical application.

## Materials and methods

### Design of the novel Z-shaped anti-rotation rod

The structure of the Z-shaped rod consists of three parts (Fig. 1A). One end of the rod is 15 mm long with a “D” shaped cross-section for anti-rotation. The other end is 35 mm long with an “O”-shaped cross-section that can be cut as needed during surgery. The two ends are connected by a “Z”-shaped part formed by two right angles, with various lengths from 0 to 8 mm. As the height of the Z-shaped rod increases, the height difference between the atlas and axis increases, leading to a greater reduction force based on the lever principle (Fig. 1B).

### Participants

Our inclusion criteria were: (1) Healthy adults; (2) No history of cervical spine diseases or surgeries; (3) Cervical CT/MRI demonstrating no developmental malformations or structural abnormalities. Finally, a 25-year-old healthy male volunteer (height： 173 cm and weight： 65 kg) was selected for the study. Our study protocol was approved by the institutional Ethics Committee of our research institution (2024012).

### Finite element model construction and validation

#### Intact cervical model

The vertebral geometry data for the bottom of the occipital bone (C0) to C7 (C0-C7) were obtained from computed tomography scans with 1-mm section thickness (Siemens, Germany). The scanned images were saved in standard Dicom format. Then we input the obtained raw files into Mimics 21.0 (Materialise, Leuven, Belgium). A 3-dimensional spine model consisting of the occipital- cervical 7 vertebrae complex was created by performing image enhancement, threshold segmentation, region growing, and Boolean operations in Mimics. The 3D model files in STL format were then imported into the reverse engineering software Geomagic Studio 2013 (Geomagic, Inc., USA) where post-processing such as denoising, relaxation, smoothing, and surfacing were carried out. The obtained 3D solid model was imported into SolidWorks 2012 (Dassault Systèmes, France) software for assembly of the vertebral body, intervertebral disc, cartilage, joint capsule, and ligaments to make the complete cervical spine model more fit to reality. Finally, the model was imported into ANSYS 2021 R1 (ANSYS Inc., USA) software to complete the meshing and assigning material properties to obtain a normal cervical spine finite element model. Material properties are specified in Table 1. The resulting spine model contained the following major components: the lower part of C0, C1-C7 vertebrae, intervertebral cartilage, and 11 spinal ligaments. The spinal ligaments included the anterior longitudinal, posterior longitudinal, supraspinous and interspinous, flavum, alar, apical ligament of the dens, anterior and posterior atlantooccipital membranes, capsular, and transverse ligaments. The vertebral bodies were meshed with tetrahedral elements. The spinal ligaments were modeled using spring elements. Linear elasticity was applied to bone, intervertebral disc, and cartilaginous structures.

**Table 1:** Material properties employed in the finite element model

Component/Materials	Young’s Modulus (MPa)	Poisson’s Ratio	Element type
Cortical bone	12000.00	0.29	C3D4
Cancellous bone	450.00	0.29	C3D4
Cartilage	10.00	0.30	C3D4
Annulus	3.00	0.45	C3D4
Ligamentum favum			
ALL			Spring element
PLL			Spring element
ISL			Spring element
SSL			Spring element
FL			Spring element
AL			Spring element
ALD			Spring element
TL			Spring element
CL			Spring element
AAM			Spring element
PAM			Spring element
Endplate	500.00	0.40	C3D4
Nucleus pulposus	1.00	0.30	C3D4
Spinal implants	110000.00	0.30	C3D4

ALL = Anterior longitudinal ligament; PLL = Posterior longitudinal ligament; ISL = Interspinous ligament; SSL = Supraspinous ligament; FL = Flavum ligament; AL = Alar ligament; ALD = Apical ligament of the dens; TL = Transverse ligament; CL = Capsular ligament; AAM = anterior atlantooccipital membrane; PAM = Posterior atlantooccipital membrane

To validate our model, we compared the ROM of the C0-C1 and C1-C2 segments of the intact finite element model with the cadaver biomechanical data from Panjabi et al. [6] and the upper cervical finite element analyses conducted by Zhang et al. [7] and Ouyang et al. [8].

#### Unstable cervical and implants models

We simulated the unstable atlantoaxial model by removing the ligaments [8, 9]. Based on the normal cervical spine model, the apical ligament of the dens, the alar ligaments and the transverse ligament were removed to simulate atlantoaxial instability. Then internal fixators including 2 PSR configurations (modeling data provided by Weigao Orthopedic Materials Co Ltd, Shandong, China) were then implanted into the unstable model.

### Calculation of reduction force for two fixation configurations

**Fig. 2:**
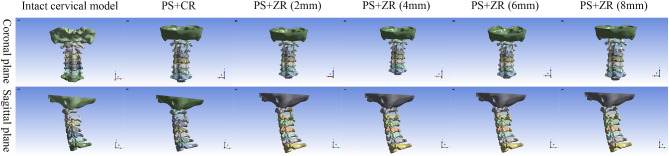
Three-dimensional reconstructed models of the intact cervical spine and different internal fixation groups ( coronal and sagittal views)

**Fig. 3:**
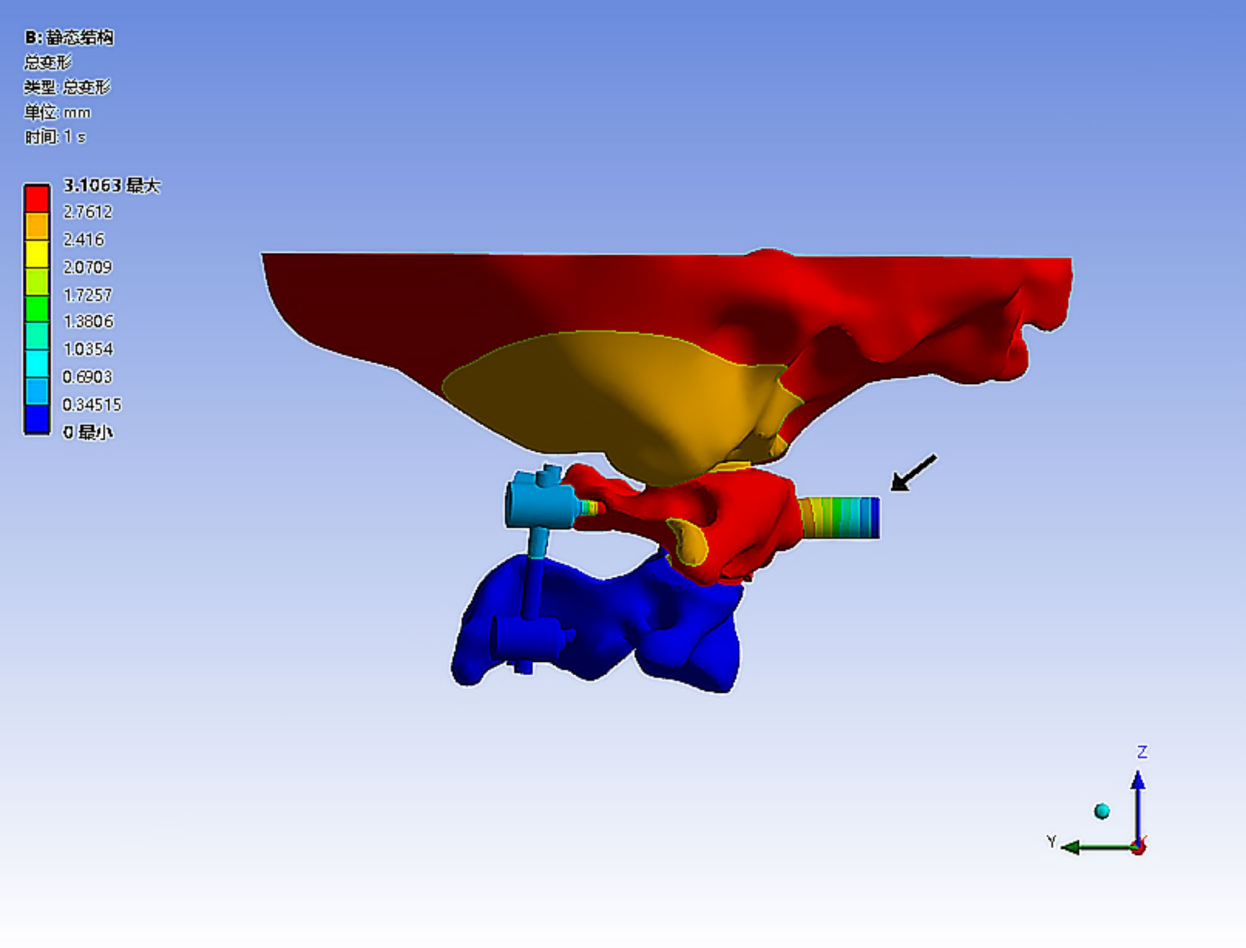
Schematic diagram of the reduction force test. (the black arrow points to the force testing module, located anterior to the anterior tubercle of the atlas)

All internal fixation groups used atlantoaxial transpedicular screw fixation, with the only difference among the groups being the connecting rods. Two fixation configurations were established with C1-C2 PSR fixation using (1) a common rod (PS-CR), and (2) Z-shaped rods with different hights (PS-ZR) (Fig. 2). We fixed the lower surface of the C3 vertebral body, defined the joint friction coefficient as 0.1, and set the friction coefficient between the screw and the cortical bone as well as the cancellous bone as 0.3. Then we added a force testing module at the anterior tubercle of the atlas and set the interface contact between the anterior tubercle of the atlas and the force testing module as bonded. We applied loading conditions to the contact surfaces. When the atlas and axis underwent relative motion, the resulting displacement was converted into elastic force by the testing module, and this force was used as the reduction force for the corresponding internal fixation configuration. The atlas was slowly pushed forward along the horizontal direction by 5 mm (while ensuring the atlantodental interval distance is greater than 3 mm) and the stress value of the testing module was recorded at this point (Fig. 3).

### Boundary and loading conditions

The inferior surface of the C3 was completely fixed in all directions. A 50 N vertical load was applied to the skull base to simulate axial gravitational loading (upright posture). A 1.5 N·m pure moment was applied on the superior surface of the occipital bone to simulate six physiological movements including flexion, extension, left lateral bending, right lateral bending, left axial rotation, and right axial rotation.

## Results

### Validation of the intact cervical model

After applying equivalent loading conditions, we compared the ROM results with the prior experimental data from Panjabi et al., Zhang et al. and Ouyang et al. This comparison revealed consistent experimental results, confirming the reliability of the finite element model (Table 2).

**Table 2:** Validation of the intact model

Segments	Motion (°)	Panjabi (1988)	Zhang et al. (2007)	Ouyang et al. (2021)	This Study
C0-C1	Flexion	3.50 ± 0.60	14.50	/	7.24
C1-C2	Flexion	11.50 ± 2.00	15.00	10.60	13.16
C0-C1	Extension	21.00 ± 1.90	13.30	/	16.11
C1-C2	Extension	10.90 ± 1.10	12.70	8.85	6.16
C0-C1	Lateral bending	5.60 ± 0.70	5.50	/	3.86
C1-C2	Lateral bending	4.00 ± 0.80	5.90	6.19	2.02
C0-C1	Axial rotation	7.90 ± 0.60	8.50	/	4.97
C1-C2	Axial rotation	38.30 ± 1.70	30.60	24.10	26.01

### ROM of C0-C3 segments in different fixation configurations

Under a load of 1.5 N·m, the ROM of each model under six motion conditions was measured. We observed that the ROM decreased sequentially from the unstable cervical spine model to the intact cervical spine model and then to the internal fixation group model. Meanwhile, in the internal fixation group, when the ROM of the C1-C2 segment changed, compensatory changes in ROM occurred in the C0-C1 and C2-C3 segments. The ROM in the instability group was significantly greater than that in the intact cervical spine model. For C1-C2, in all measured directions, the ROM in the atlantoaxial instability model increased by at least 50% compared to the intact group’s cervical spine model. Flexion was the most significantly affected, increasing by 22°, followed by extension and axial rotation, which increased by an average of approximately 15°, while lateral bending increased bilaterally by an average of about 10°.

After adding internal fixations to the instability model, the ROM subsequently changed. For the C1-C2 segment, regardless of the PS-CR or PS-ZR group, the ROM in all directions significantly decreased by more than 95%. For the C0-C1 segment, the ROM in each fixation group also decreased in all directions, but the impacts on lateral bending and rotational movements were significantly greater than those on flexion-extension movements. Interestingly, we found that for the C2-C3 segment, the trend in the fixation group was completely opposite. Both ranges of extension and lateral bending movements decreased to varying degrees, while the ROM in flexion and left-right rotational movements increased, with rotational movements increasing by an average of 6–7°. Meanwhile, regardless of the fixation configuration applied, the differences in the ROM among the fixation groups were less than 5% in all six movement directions (Table 3).

**Table 3:** Comparison of the ROM in different models

Model	Flexion (°)	Extension (°)	Left Lateral bending (°)	Right lateral bending (°)	Left axial rotation (°)	Right axial rotation (°)
**Intact model**						
C0-C1	7.24	16.11	3.81	3.90	4.97	4.96
C1-C2	**13.16**	**6.16**	**2.07**	**1.96**	**25.97**	**26.05**
C2-C3	**3.20**	**2.06**	4.10	3.90	**1.39**	**1.38**
**Instability model**						
C0-C1	9.90	21.14	6.60	4.17	5.01	4.75
C1-C2	**35.02**	**22.43**	**12.87**	**11.67**	**39.02**	**40.45**
C2-C3	3.17	2.06	4.05	3.84	1.38	1.37
**PS with CR**						
C0-C1	6.63	7.05	0.44	0.47	0.74	0.83
C1-C2	**0.32**	**0.26**	**0.15**	**0.14**	**0.51**	**0.56**
C2-C3	**5.11**	**2.07**	3.08	2.93	**8.15**	**8.69**
**PS with ZR (2 mm)**						
C0-C1	6.63	7.05	0.44	0.46	0.75	0.83
C1-C2	**0.32**	**0.26**	**0.15**	**0.14**	**0.50**	**0.55**
C2-C3	**5.11**	**2.07**	3.08	2.29	**8.15**	**8.69**
**PS with ZR (4 mm)**						
C0-C1	6.63	7.05	0.44	0.46	0.75	0.83
C1-C2	**0.33**	**0.24**	**0.17**	**0.16**	**0.51**	**0.56**
C2-C3	**5.11**	**2.07**	3.08	2.29	**8.15**	**8.69**
**PS with ZR (6 mm)**						
C0-C1	6.64	7.05	0.44	0.46	0.75	0.83
C1-C2	**0.34**	**0.24**	**0.18**	**0.16**	**0.51**	**0.56**
C2-C3	**5.11**	**2.07**	3.08	2.29	**8.15**	**8.69**
**PS with ZR (8 mm)**						
C0-C1	6.64	7.05	0.44	0.46	0.75	0.83
C1-C2	**0.35**	**0.24**	**0.19**	**0.17**	**0.52**	**0.57**
C2-C3	**5.11**	**2.07**	3.08	2.29	**8.15**	**8.69**

PS = pedicle screw; CR = common rod; ZR = Z-shaped rod

### Comparison of reduction forces

We found that the reduction force of the Z-shaped rod could be several times greater than that of the common rod as the fulcrum height increased. Using the force feedback from the force testing module, we measured the reduction force magnitude in different internal fixation groups. When the conventional PS-CR fixation was used, the reduction force reached 24.079 N. When replaced with 2 mm PS-ZR fixation, the reduction force reached 40.188 N, which was 1.67 times that of the common rod. For different sizes of Z-shaped rods, the reduction force increased by approximately 15 N for every 2 mm increase in height. When we used the 8 mm PS-ZR configuration, the reduction force reached 88.544 N, which was 2.20 times that of the 2 mm PS-ZR configuration and 3.68 times that of the PS-CR configuration (Table 4).

**Table 4:** Comparison of reduction forces and peak stress values

Model	Reduction Forces (*N*)	Flexion (*N*)	Extension (*N*)	Left Lateral bending (*N*)	Right lateral bending (*N*)	Left axial rotation (*N*)	Right axial rotation (*N*)
PS with CR	**24.08**	154.24	100.99	90.877	96.79	224.23	241.29
PS with ZR (2 mm)	**40.19**	158.20	102.46	160.03	115.26	233.04	251.90
PS with ZR (4 mm)	**56.24**	162.71	104.04	147.19	131.25	234.06	253.81
PS with ZR (6 mm)	**72.37**	161.32	110.04	157.13	139.09	235.31	258.88
PS with ZR (8 mm)	**88.54**	162.29	109.64	131.51	137.91	238.39	262.85

PS = pedicle screw; CR = common rod; ZR = Z-shaped rod

### Stress distribution analysis of implants and vertebral bodies

**Fig. 4:**
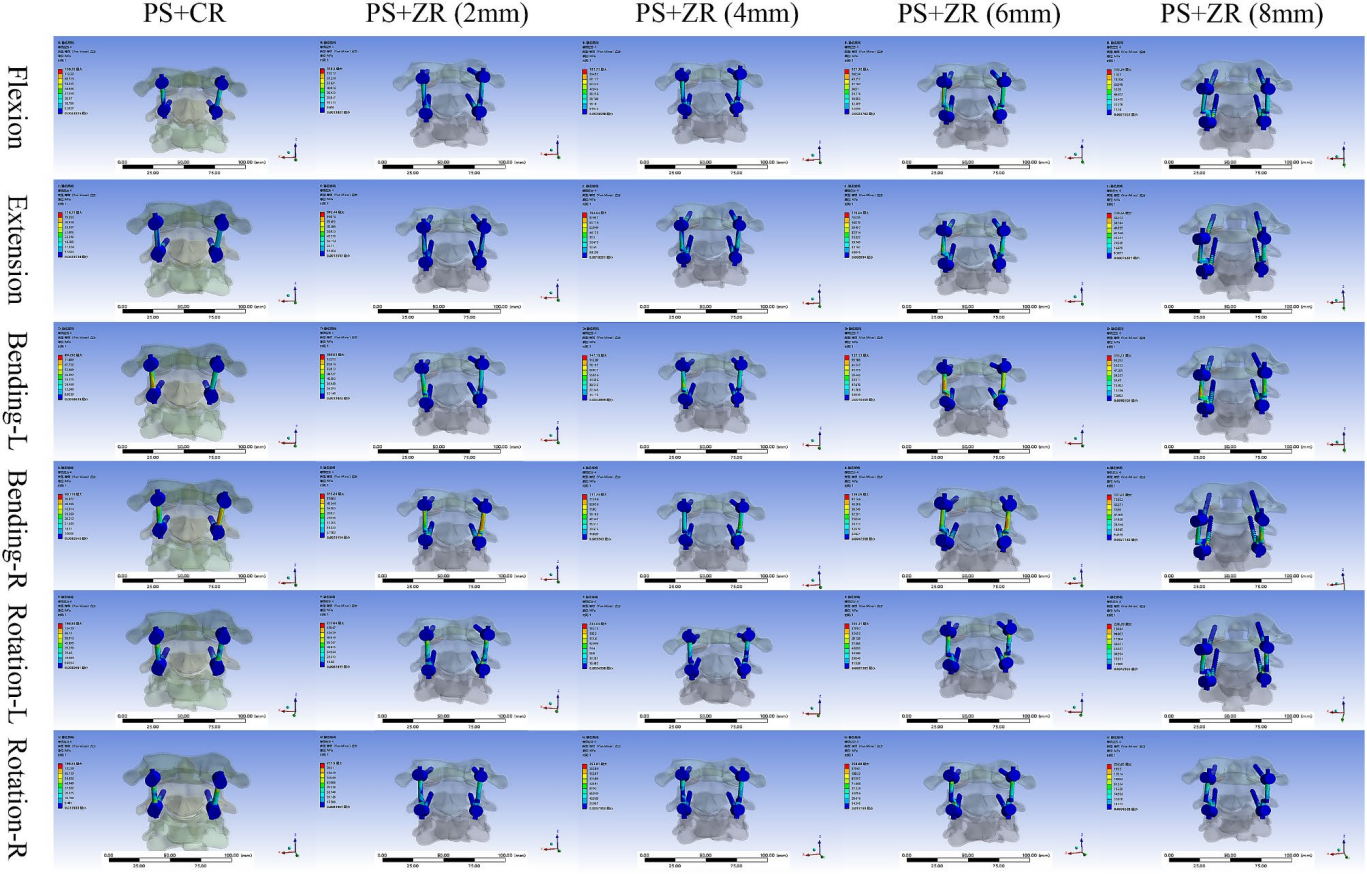
Stress distribution nephograms of the vertebral body-implants contact surfaces (PS + CR vs. PS + ZR) in six motion states. PS = pedicle screw; CR = common rod; ZR = Z-shaped rod. Bending-L = Left lateral bending; Bending-R = Right lateral bending; Rotation-L = Left rotation; Rotation-R = Right rotation

The von Mises stress contour plot showed that the stress distribution areas of each fixation technique were comparable (Fig. 4). For each internal fixation configuration, the peak stress occurred during left and right axial rotation, with the minimum value being 241.29 N for the common rod configuration and the maximum value being 262.85 N for the 8 mm Z-shaped rod configuration. As the fulcrum of the Z-shaped rod continued to increase in height, the maximum stress borne by the PS-ZR system gradually increased, with approximate increases of 10 N, 40 N, and 20 N during flexion-extension, right lateral bending, and axial rotation, respectively. However, for left lateral bending, although the stress also increased, the changes were not regular (Table 4).

**Fig. 5:**
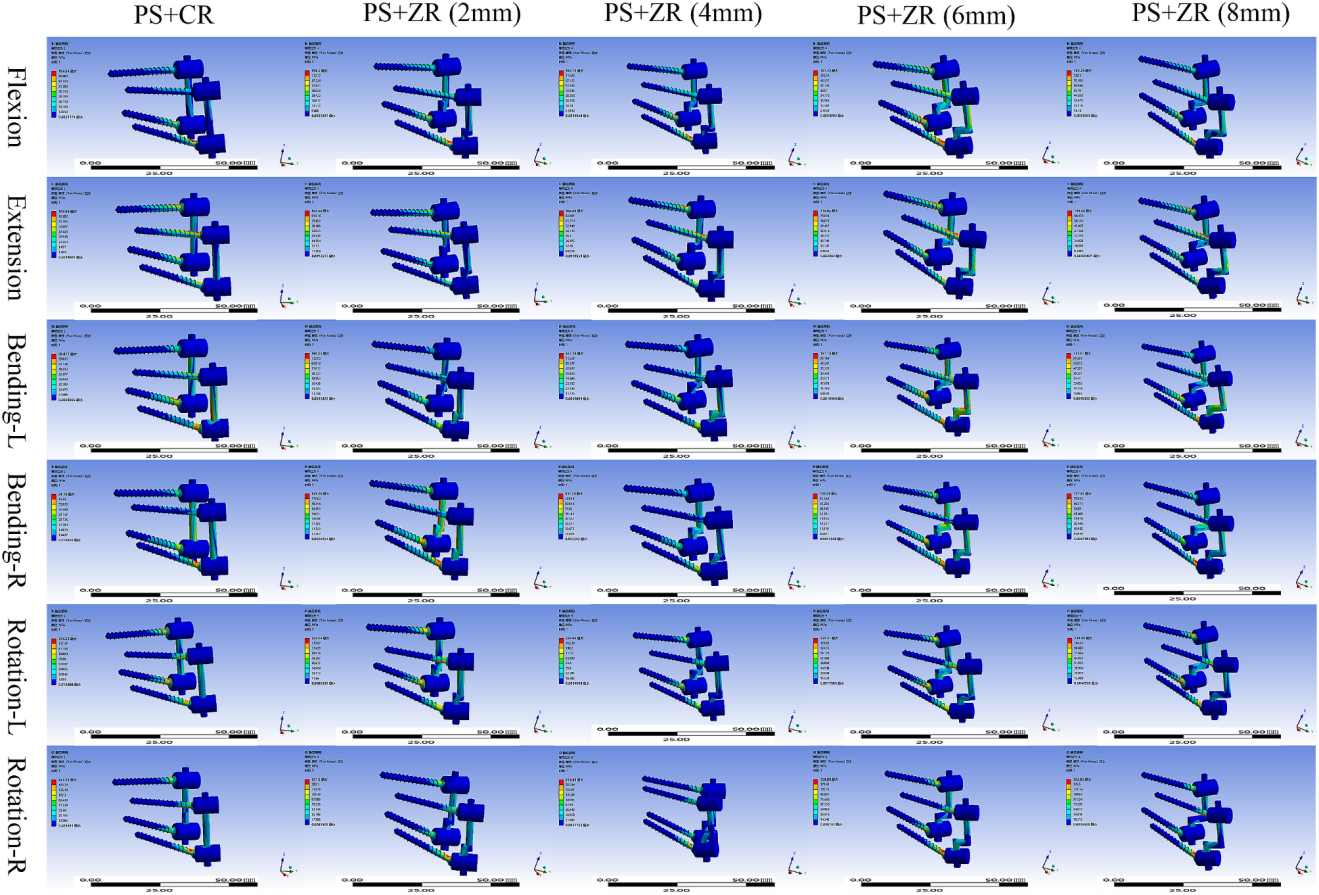
Stress distribution nephograms of implants (PS + CR vs. PS + ZR) in six motion states. PS = pedicle screw; CR = common rod; ZR = Z-shaped rod; Bending-L = Left lateral bending; Bending-R = Right lateral bending; Rotation-L = Left rotation; Rotation-R = Right rotation

Regarding the areas of stress concentration, we found that for vertebral bodies, the maximum stress was concentrated at the connection interface between the screw and the bone. For the pedicle screw, the maximum stress on the screws was mainly concentrated at the junction between the screw threads and the tail cap. For the screw-rod interface, the maximum stress on the PS-CR system was mainly concentrated at the connection between the rod and the screw nut while the maximum stress on the PS-ZR system was concentrated at the transition part of the “Z” shape (Fig. 5).

## Discussion

In this study, we constructed an atlantoaxial instability model through finite element analysis, then evaluated and compared the reduction force and stress distribution between the PS-CR system and the PS-ZR system. As shown in the results, the novel Z-shaped anti-rotation rod demonstrates excellent biomechanical properties. It achieves a multi-fold increase in reduction force, eliminates the need for release surgery and saves time in rod bending, offering significant clinical value.

Atlantoaxial dislocation can be specifically classified into reducible, refractory, and irreducible dislocations [10]. For reducible dislocations, reduction is relatively easy, and posterior screw-rod fixation and fusion are often directly performed. For irreducible dislocations, due to the presence of bone connective fusion between the atlas and axis, direct reduction is not possible. Generally, the surgical scope of the release procedure should be determined based on the extent of bone fusion (whether it is focal or extensive). For refractory dislocations, it is challenging to achieve satisfactory reduction solely relying on heavy skull traction and the pulling force of the existing posterior screw-rod internal fixation system. To address this issue, potential solutions include enhancing the reduction capacity of the internal fixation system or diminishing the resistance to reduction between the atlas and axis. While a multitude of studies have demonstrated the efficacy of anterior or posterior soft tissue release in managing refractory atlantoaxial dislocations [11–14], it is important to weigh the potential drawbacks. The release procedure not only extends operative duration, elevates the risks of hemorrhage and infection but also results in the disruption of extensive anatomical structures [15, 16]. These factors collectively heighten the likelihood of inadvertent damage to the spinal cord, nerves, and vasculature, thereby substantially amplifying the risk of complications associated with the surgery. In addition, for our junrior surgeons, performing a high cervical release surgery combined with posterior internal fixation represents a formidable challenge, marked by a steep and prolonged learning curve. A meta-analysis conducted by Guan et al. [17] also indicated that for atlantoaxial dislocation, release surgery may not be necessary as direct posterior reduction showed less surgical trauma and a shorter operation time with comparable efficacy. Therefore, if good reduction can be simply achieved by enhancing the reduction capability of the posterior screw-rod system, it would yield numerous clinical advantages. These include diminishing the proportion of patients necessitating release surgery, reducing the intricacy and complexity of atlantoaxial surgery, broadening the scope of posterior surgery applicability, and promoting clinical dissemination and implementation.

As demonstrated earlier, the current posterior screw-rod system frequently employs a pre-bent cylindrical connecting rod to augment the height disparity between the atlas and axis, thereby bolstering the reduction capability. However, the reduction process is often compromised by the propensity of the connecting rod to rotate within the screw slot, leading to a diminution and inadequate transmission of the reduction force. Previous studies have predominantly focused on refining surgical techniques to enhance reduction outcomes, including improvements in release surgery and intra-articular traction [16, 18, 19]. In recent years, some researchers have also proposed improvements from the perspective of surgical instruments. He et al. [20]invented a novel head-neck fixation and traction device for patients with irreducible atlantoaxial dislocation, which can improve surgical outcomes and quality of life. Ma et al. [21] engineered a novel posterior reduction forceps for atlantoaxial dislocation that can assist the simple posterior screw-rod system in the treatment of irreducible atlantoaxial dislocation to avoid the release in anterior or posterior approach and reduce the complexity of surgery. In terms of implant improvements, Ouyang et al. [8] first reported a new type of horizontal screw-screw crosslink (hS-S CL) and confirmed by finite element analysis that hS-S CL can provide a more stable architecture for the internal fixation. However, there is no relevant research on connecting rod optimization. Our innovative design of the Z-shaped anti-rotation reduction rod has paved the way for this field. “D”-shaped cross-section can fully fit the screw slot, theoretically avoiding the issue of rod rotation, while the Z-shaped corner structure increases the reduction height difference between the atlas and axis, enhancing the reduction capability of the PSR system.

Meanwhile, previous finite element studies on the upper cervical spine, when analyzing the ROM, mostly focused on changes in the C1-C2 segment [8, 22, 23], while only a few studies addressing the ROM changes in the adjacent atlanto-occipital joint and C2-C3 joint [24]. Considering that the occipitocervical junction complex is the region with the greatest spinal mobility, its motion mechanics are highly intricate. Our study confirmed that when atlantoaxial internal fixation is performed, compensatory changes in the ROM occur in both the atlanto-occipital joint and the C2/3 joint. Particularly in the C2/3 segment, due to the downward transmission of overall forces, axial rotation significantly increases, which may be a risk factor for postoperative adjacent segment disease (ASD) and warrants attention. Since the reduction force of the PSR system cannot be directly measured and no prior research available could be used for reference, we innovatively designed an atlantoaxial force testing module and derived the internal fixation reduction force by pulling the atlas in reverse. This method circumvents the complex modeling conditions required for direct measurement of reduction forces while maximally simulating the surgical process of lifting and reducing dislocated joints. The results confirmed that this method effectively reflected the differential reduction capabilities between the PS-CR and the PS-ZR, which can serve as an important reference for future research. Finally, this novel Z-shaped anti-rotation rod not only proves clinical application values in atlantoaxial dislocation surgery but also holds significant reference value for future improvements in posterior reduction and fixation systems in cases of lower cervical dislocations and thoracolumbar spondylolisthesis.

This study has certain limitations. First, the three-dimensional finite element model in our study was reconstructed based on CT scan data obtained from a representative human body. Due to inter-individual variations, even with meticulous processing during modeling, it is challenging to fully replicate in vivo conditions. Second, to simplify computational complexity and reduce modeling time costs, muscles and soft tissues were not included in our model, which makes the model unable to fully simulate the in vivo conditions. Future studies should incorporate in vivo validation to confirm the biomechanical advantages observed in finite element simulations. Additionally, incorporating muscle and soft tissue models could further enhance accuracy. Finally, for the testing of reduction force, the reduction force testing relied on an innovative but indirect method, and all data were instantaneous biomechanical data. This method neglected the attenuation caused by force transmission in the internal fixation system, which may result in measured data being greater than the actual reduction force.

## Conclusion

The novel Z-shaped anti-rotation rod demonstrates superior biomechanical properties compared to the common rod in finite element analysis. Its enhanced reduction force may reduce the need for release surgery and save surgery time, making it a promising advancement in atlantoaxial dislocation management. Further clinical validation is warranted.

## Data Availability

No datasets were generated or analysed during the current study.
